# Middle-aged mice show delayed and destabilized food-anticipatory circadian activity under restricted feeding

**DOI:** 10.1016/j.jphyss.2026.100071

**Published:** 2026-04-02

**Authors:** Sachi N. Ohno, Nana N. Takasu, Hitoshi Uchida, Takahiro J. Nakamura, Wataru Nakamura, Mitsutaka Sugimura

**Affiliations:** a Department of Dental Anesthesiology, Graduate School of Medical and Dental Sciences, Kagoshima University, Kagoshima 890-8544, Japan; b Department of Anesthesiology, Aso Iizuka Hospital, Iizuka, Fukuoka 820-8505, Japan; c Department of Oral Chrono-Physiology, Graduate School of Biomedical Sciences, Nagasaki University, Nagasaki 852-8588, Japan; d Laboratory of Animal Physiology, School of Agriculture, Meiji University, Kawasaki, Kanagawa 214-8571, Japan

**Keywords:** Aging, Circadian rhythm, Food-anticipatory activity, Restricted feeding

## Abstract

Mice are nocturnal and normally feed during the night; however, timed daily feeding can markedly alter behavioral and physiological rhythms. A well-known example is food-anticipatory activity (FAA), characterized by increased locomotor activity before feeding. In this study, we examined age-related effects on FAA under temporally restricted feeding (RF). Male C57BL/6 J mice aged 12 weeks and 54 weeks were maintained under a 12:12 light–dark cycle and subjected to a 4-h daily RF schedule. Wheel-running activity, feeding timing, intake, and body weight were monitored. Under ad libitum conditions, both age groups exhibited nocturnal activity and feeding. During RF, FAA emerged significantly earlier in young mice than in middle-aged mice. After returning to ad libitum feeding, daytime activity gradually merged into nocturnal rhythms, although some middle-aged mice showed unstable patterns. These results indicate that FAA reflects a circadian system distinct from the suprachiasmatic nucleus and that aging delays and destabilizes its expression.

## Highlights

• Middle-aged mice show delayed emergence of food-anticipatory activity.
• Coupling between food-entrainable oscillator and SCN may be weakened.
• Circadian anticipation becomes unstable in middle-aged mice.
• Circadian plasticity in feeding-related behavior is reduced.

## Introduction

Proper phase relationships among physiological and behavioral rhythms are crucial for maintaining optimal health and adaptation to the environment. Under ad libitum conditions, feeding behavior in nocturnal rodents is concentrated during the dark phase, accounting for more than 80% of total daily intake [1]. In contrast, mice carrying mutations in core clock genes exhibit attenuated feeding rhythms and develop obesity and metabolic disorders [2]. The rhythm of feeding behavior depends largely on the suprachiasmatic nucleus (SCN), the principal circadian pacemaker, because feeding rhythmicity disappears when the SCN is lesioned and feeding becomes evenly distributed over 24 h [3].

When food availability is restricted to a specific time of day, many spontaneous behaviors gradually shift their phase to align with the feeding time after a transient period of several days. Richter [4] was among the first to describe such phase adjustments in feeding-related behavior, laying the foundation for later studies on food-entrainable rhythms. A marked increase in locomotor activity before feeding—termed food-anticipatory activity (FAA)—represents a behavioral manifestation of food-entrainable circadian oscillators [5], [6], [7]. FAA is not simply a masking effect in response to external cues but an entrained oscillation that persists under constant conditions. It is also observed in physiological variables such as plasma corticosterone levels [8] and remains expressed even after ablation of the SCN [9], [10], indicating that it is generated by a circadian oscillator independent of the SCN. Moreover, restricted feeding exerts a stronger influence than light on circadian rhythms of hepatic enzyme activity, and time-restricted feeding can prevent obesity and metabolic syndrome even in clock-deficient mice [11].

Aging alters circadian rhythms in both animals and humans, accompanied by reduced output and weakened synchrony of the SCN [12]. Age-related deterioration of circadian organization results in dampened behavioral rhythmicity, increased fragmentation, and slower re-entrainment after phase shifts [13], [14]. In rodents, aging is also associated with reduced amplitude of wheel-running rhythms [15], [16] and attenuated adaptability to environmental changes such as light-dark (LD) transitions [17]. Although the mechanisms of aging in the SCN have been studied extensively, much less is known about how aging influences food-entrained circadian rhythms.

In the present study, we examined wheel-running activity rhythms in young (12-week-old) and middle-aged (54-week-old) C57BL/6 J mice during and after restricted feeding and analyzed associated changes in food intake and body weight. Our results demonstrate that age-related changes delay the emergence of FAA, supporting the concept of an anticipatory circadian system that can be uncoupled from the master pacemaker and suggesting that weakening of internal synchrony may underlie altered metabolic and behavioral adaptation.

## Materials and methods

### Animals and ethics

Male C57BL/6 J mice aged 12 weeks and 54 weeks were maintained under a 12:12 light–dark cycle and subjected to a 4-h daily restricted feeding (RF) schedule. All procedures were approved by the Institutional Animal Care and Use Committee of Nagasaki University (protocol numbers #1809181479–2 and #2308231890) and were conducted in accordance with institutional guidelines for animal experimentation.

### Wheel-running activity measurements

Wheel-running activity was recorded as previously described [18]. Briefly, wheel revolutions were detected every minute using a magnetic sensor (Hamlin 59070–010) attached to a 12-cm stainless-steel wheel and automatically stored via ClockLab software (Actimetrics, Wilmette, IL). Cages were placed in light-tight, ventilated boxes (≈80 lux at cage level, white LED source ELG-01B(W); ELPA, Osaka, Japan) under the same environmental conditions as the animal room. Activity was continuously monitored throughout the experiment, and actograms were generated in 6-min bins for individual analysis. Animals showing persistently low or absent wheel-running activity during the recording period were excluded from the analysis.

### Experimental protocol for body weight and food consumption

Mice were provided regular chow (LABO MR Stock, Nosan, Yokohama, Japan) placed on the wire-top of each cage. Wheel-running activity was first recorded for 7 days under ad libitum feeding. Mice were then food-deprived for 30 h and subsequently subjected to a 4-h restricted feeding (RF) schedule (ZT6–ZT10) for 4 weeks. This feeding schedule was selected based on previous studies demonstrating that a 4-h RF protocol reliably induces robust food-anticipatory activity [19]. After RF, animals were returned to ad libitum feeding for 2 weeks under the same LD cycle. Body weights were measured daily just before ZT6, except on the fasting day. Under RF conditions, food was manually provided and removed at the designated times, and food consumption was measured as the difference in pellet weight before and after feeding. For the second experimental series, after 3 weeks of recovery, both groups (young-adult, 20 weeks old; middle-aged, 64 weeks old; n = 7 per group) were re-exposed to the same RF regimen (1 week ad libitum, 30-h fasting, 4 weeks of 4-h RF under LD). Thereafter, mice were released into constant darkness (DD) while being returned to ad libitum feeding with excess food pellets available. Wheel-running activity was continuously recorded throughout both experimental series.

### Data analyses

Activity data were analyzed using ClockLab Analysis software (v2.72, Actimetrics, USA). The emergence of FAA was determined by visual inspection of three blinded examiners. Statistical comparisons between two groups were made using Student’s *t*-tests. For comparisons involving three or more groups, one-way ANOVA followed by Dunnett’s post hoc test was applied. Data are presented as means ± SEM, and differences were considered significant at p < 0.05.

## Results

### Body weight, food intake, and wheel-running activity before restricted feeding

**Fig. 1:**
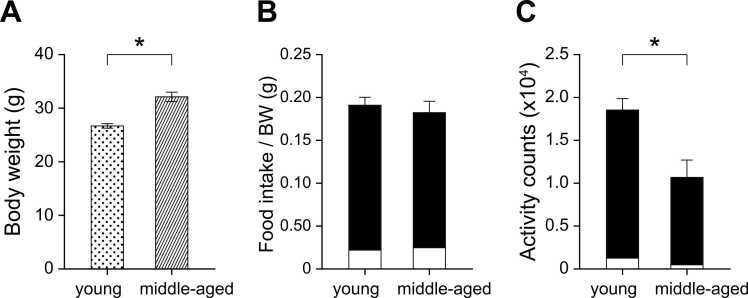
Altered body weight, food intake, and wheel-running activity in young-adult and middle-aged mice under ad libitum feeding. (A) Comparison of body weight (young-adult, n = 8, dotted bar; middle-aged, n = 8, hatched bar) measured at ZT6. (B) Diurnal rhythm of food intake. Mice were maintained under ad libitum feeding, and food intake (g per body weight) was recorded during the light (white bars) and dark (black bars) periods. (C) Diurnal rhythm of wheel-running activity counts during the light and dark periods. All values represent group means ± SEM. Asterisks denote significant differences (p < 0.01).

Fig. 1 shows comparisons of body weight (Fig. 1A), food intake relative to body weight (Fig. 1B), and wheel-running activity (Fig. 1C) in young-adult and middle-aged mice under ad libitum feeding. The mean body weight of middle-aged mice (32.1 ± 0.9 g; mean ± SEM, n = 8) was significantly higher than that of young-adult mice (26.7 ± 0.4 g, n = 8; t = −5.59, p < 0.01; Fig. 1A). However, there were no significant differences in daily food intake relative to body weight between the two groups (t = 0.59, p = 0.30; Fig. 1B). The proportion of nocturnal feeding was 89.2 ± 3.7% in young and 86.3 ± 2.6% in middle-aged mice, indicating a consistent nocturnal preference for food intake in both groups. Both groups displayed minimal activity during the light phase and initiated robust wheel-running activity after lights-off under ad libitum conditions. The mean daily wheel-running counts in young-adult mice (1.85 ± 0.13 × 10⁴ counts/day) were significantly higher than those of middle-aged mice (1.07 ± 0.20 × 10⁴ counts/day; t = 3.26, p < 0.01; Fig. 1C). Nocturnal activity rates were 92.9 ± 2.7% and 93.7 ± 1.8% in young and middle-aged mice, respectively, confirming that both age groups maintained clear nocturnal activity patterns.

### Anticipation in wheel-running activity

**Fig. 2:**
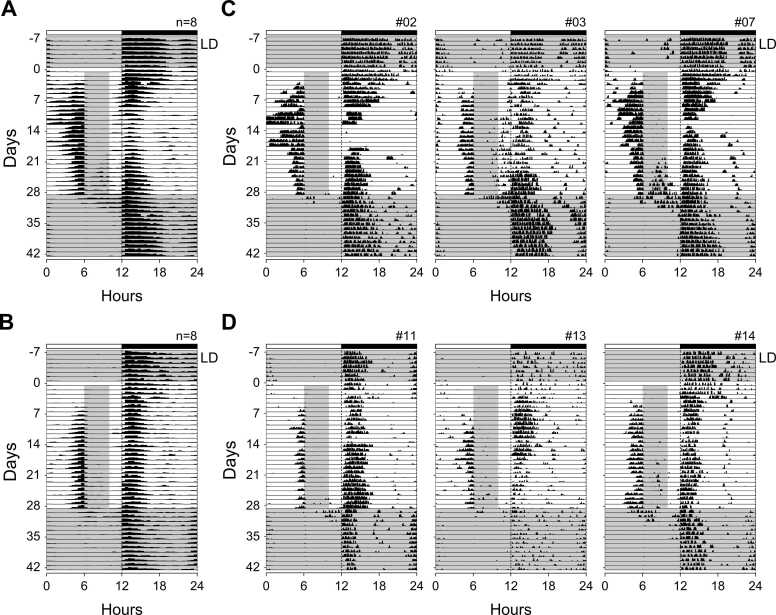
Wheel-running activity of mice kept under a 24-h LD cycle and alternately fed ad libitum or for 4 h per day. (A, B) Average wheel-running profiles of young-adult (A) and middle-aged (B) mice (n = 8 per group) plotted in 30-min bins. (C, D) Representative single-animal actograms (6-min bins) from young-adult (C) and middle-aged (D) mice. White and black bars at the top indicate light and dark phases, respectively. The gray area denotes the 4-h feeding period (ZT6–ZT10). Each horizontal line represents 1 day; day 0 corresponds to the start of restricted feeding following 30-h fasting.

**Fig. 3:**
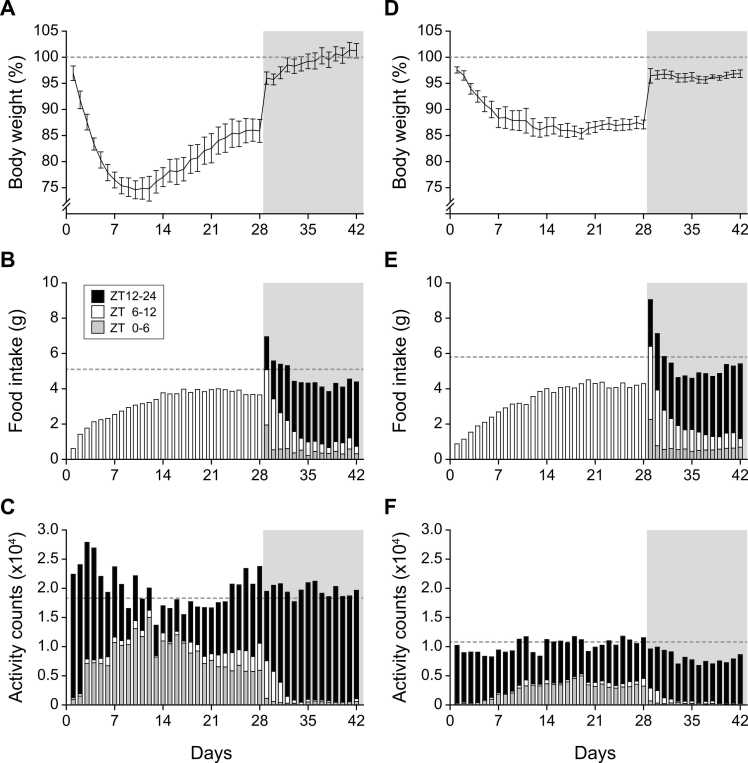
Changes in body weight, food intake, and wheel-running activity during and after restricted feeding (RF). Daily values for young-adult (A–C) and middle-aged (D–F) mice are shown (n = 8 mice per group). Day 0 marks the beginning of RF following 30-h fasting; gray-shaded areas indicate ad libitum feeding periods. Dotted horizontal lines denote baseline (day −1) levels. Histograms show data for the first half of the light period (ZT 0–6, gray), second half (ZT 6–12, white), and dark period (ZT 12–24, black).

Fig. 2 illustrates representative actograms and averaged profiles of wheel-running activity in young-adult and middle-aged mice under a 24-h LD cycle during alternating ad libitum and restricted-feeding (RF) schedules. Day 0 denotes the initial 30-h fasting period before timed feeding began. During ad libitum feeding (days −7 to −1), both age groups showed little activity during the light phase. After fasting, mice were fed for 4 h each day (ZT6–ZT10) for 28 days. In the first few days of RF, wheel-running remained predominantly nocturnal, but a distinct increase in daytime activity appeared a few days later as FAA. This anticipatory component emerged significantly earlier in young-adult mice (3.3 ± 1.0 days) than in middle-aged mice (7.4 ± 4.7 days; p < 0.05). The onset of FAA marked a gradual shift of activity from the nocturnal to the daytime phase (Fig. 2; see also Fig. 3C, F). Concomitantly, the pre-light-on peak of nocturnal activity diminished, particularly in young-adult mice, indicating a redistribution of circadian activity toward the feeding time. Following the termination of RF on day 29 (return to ad libitum feeding at ZT0), FAA persisted for several days in both age groups before merging back into the nocturnal component. The persistence of daytime activity was evident in individual actograms—for example, animals #03 and #07 among the young-adult group and #11 and #14 among the middle-aged group—demonstrating that the FAA oscillator remained active for a short transitional period after food timing was removed.

### Body weight, food intake, and wheel-running activity during restricted feeding

As restricted feeding (RF) began following a 30-h fast, both young-adult and middle-aged mice lost several percent of body weight (3.1 ± 1.4% and 2.4 ± 0.6%, respectively). On the first RF day, food intake in the young group (0.61 ± 0.13 g) corresponded to the daytime portion under ad libitum feeding (0.60 ± 0.23 g; about 12% of total daily intake). During the first week, young-adult mice continued to lose weight to ∼75% of baseline, then gradually recovered and stabilized around 85% after three weeks of timed feeding (Fig. 3A). Food intake increased in parallel, reaching ∼75% of baseline by day 14 and remaining stable thereafter (Fig. 3B). Middle-aged mice also lost weight after the onset of RF but at a slower rate. On day 8, mean body weight decreased to 75.4 ± 1.5% in young-adult mice versus 88.4 ± 2.1% in middle-aged animals. Thereafter, middle-aged mice maintained > 85% of baseline weight throughout the 28-day RF period (Fig. 3D). When averaged over the last 7 days, body weight was 85.3 ± 2.4% in young and 87.1 ± 1.0% in middle-aged mice. Across the 28-day RF and subsequent 14-day recovery, daily wheel-running counts showed modest fluctuations without significant differences from baseline except on day 3 in young-adult mice (p = 0.037, Dunnett’s post hoc test). However, activity restricted to the pre-feeding phase (ZT 0–6) increased markedly after day 3 in young-adult and after day 10 in middle-aged mice, consistent with the timing of FAA formation (Figs. 2 and 3C, F).

### Rebounded weight gain and recovery of nocturnal feeding and activity after resumption of ad libitum feeding

Upon termination of RF on day 29 (return to ad libitum feeding at ZT 0), both age groups exhibited transient hyperphagia and reduced activity. Young-adult mice consumed 1.95 ± 0.25 g during ZT 0–6 (Fig. 3B) while wheel-running counts dropped from 0.59 ± 0.16 × 10⁴ to 0.11 ± 0.06 × 10⁴ counts (Fig. 3C). Consequently, they regained 2.7 ± 0.3 g and reached 96.0 ± 1.2% of pre-RF body weight. Middle-aged mice showed comparable responses: food intake 2.27 ± 0.37 g (ZT 0–6), activity reduced from 0.32 ± 0.10 × 10⁴ to 0.07 ± 0.05 × 10⁴ counts (Fig. 3E,F), and weight rebound 3.0 ± 0.4 g (96.4 ± 1.4% of pre-RF). After this rebound, both groups initially maintained a daytime preference for feeding (ZT 6–12) corresponding to the former RF period, while gradually restoring nocturnal feeding. Young-adult mice reverted to exclusive nocturnal intake by day 37 (ninth day of ad libitum feeding), whereas middle-aged mice continued partial daytime feeding longer, indicating delayed recovery of nocturnal rhythmicity.

### Persistence of daytime activity after the termination of restricted feeding

**Fig. 4:**
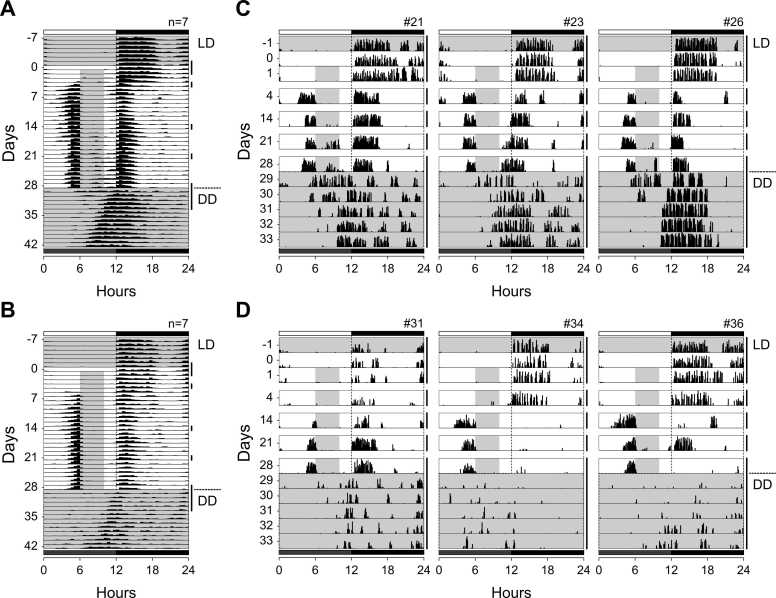
Persistence of daytime activity after the termination of restricted feeding and release into constant darkness. (A, B) Group-averaged actograms of wheel-running activity in young-adult (A; n = 7) and middle-aged (B; n = 7) mice, plotted in 30-min bins. (C, D) Representative actograms from individual young-adult (C) and middle-aged (D) mice. After 4-h restricted feeding under a LD cycle, animals were returned to ad libitum feeding and released into DD (indicated at the right side of actograms). Activity profiles shown correspond to days −1, 0 (30 h fasting), 1, 4, 14, 21, 28, and 29–33, illustrating temporal changes before, during, and after RF. Gray shading indicates the 4-h feeding period (ZT 6–10). Each horizontal line represents 1 day. See also Fig. 2 for actogram conventions.

To examine whether the daytime food-anticipatory component persisted independently of the LD cycle, both young-adult and middle-aged mice were subjected to a second series of restricted feeding (RF) followed by release into constant darkness (DD) upon returning to ad libitum feeding on day 29. One animal from each group was excluded due to markedly reduced wheel-running activity. As observed in the first series under LD, young-adult mice exhibited a persistent daytime activity component for several days after the transition to ad libitum feeding (Fig. 4A). This component gradually merged into the free-running rhythm with a delay of several days, indicating that the anticipatory oscillator continued to operate transiently in the absence of external light cues. Similar patterns were consistently observed in all young-adult mice, three of which are shown in Fig. 4C. These results confirm that feeding-associated rhythms possess circadian properties and can dissociate from nocturnal behavior rhythms governed by the SCN. Middle-aged mice showed a comparable but weaker persistence of daytime activity (Fig. 4B, D). Some individuals exhibited transient arrhythmicity during the DD phase, and the re-establishment of nocturnal rhythmicity was slower and less stable than in young-adult mice. These findings suggest that age-related changes attenuate the strength and coherence of food-entrained oscillators responsible for sustaining anticipatory rhythms under constant conditions.

## Discussion

### Aging and food-entrainable circadian coupling

The present findings demonstrate that FAA induced by restricted feeding is not a transient response to an external cue but represents the expression of a circadian rhythm distinct from that governed by the SCN. Moreover, the timing and stability of this rhythm differed between young-adult and middle-aged mice. It should be noted that middle-aged mice differed from young mice not only in age but also in body weight and likely absolute food intake. Although food intake normalized to body weight was similar between groups, differences in metabolic status or body composition may have contributed to the observed changes in FAA. Therefore, the present findings cannot fully dissociate the effects of aging per se from those of altered metabolic conditions, and this should be considered as a limitation of the study.

In particular, daytime activity corresponding to FAA in middle-aged mice failed to merge into the nocturnal component, as typically observed in young adults. This observation suggests that the coupling between the food-entrainable oscillator (FEO) and the SCN may be weakened with aging. Such age-related attenuation of coupling may cause partial desynchronization between the SCN-driven nocturnal rhythm and the FEO-driven diurnal component, thereby reducing the system’s ability to re-establish temporal coherence of behavior after restricted feeding. However, it should be noted that the present study is based primarily on behavioral measurements, and therefore does not directly assess the underlying neural or molecular mechanisms. Thus, the interpretation regarding FEO-SCN coupling should be considered as an inference based on behavioral evidence rather than a direct demonstration of circadian network interactions.

### Persistence and circadian nature

The persistence of daytime activity after termination of restricted feeding and release into DD provides clear evidence that the FAA rhythm possesses circadian properties rather than being a transient, stimulus-driven response. In young-adult mice, the daytime FAA component persisted for several days following the end of RF and gradually merged into the nocturnal free-running rhythm (Fig. 4A, C). This gradual transition parallels the phenomenon observed in other circadian oscillators once entrainment cues are removed, demonstrating that FAA is generated by an internal oscillatory process. In contrast, middle-aged mice exhibited a prolonged or incomplete merging of the daytime component, and in some individuals, transient arrhythmicity appeared during the post-RF period (Fig. 4B, D). Such instability indicates a reduced capacity of the aging circadian system to re-establish phase coherence between oscillators once external time cues are withdrawn.

The persistence of FAA under constant conditions has long been considered a hallmark of circadian regulation. Indeed, SCN-ablated rodents retain FAA that free-runs for a few cycles after the cessation of RF in mice [19], and similar oscillatory persistence has been reported for pre-feeding elevations in plasma corticosterone in rats [20]. These observations, together with our present findings, support the conclusion that FAA is governed by a food-entrainable oscillator capable of sustaining rhythmic output independently of the SCN. Previous studies using genetically manipulated models lacking specific clock genes have provided important insights into the mechanisms underlying food-anticipatory rhythms. In such models, food-anticipatory activity can persist even in the absence of functional SCN-driven circadian rhythms, supporting the existence of a distinct food-entrainable oscillator system. These findings suggest that the relationship between the SCN and FEO is not hierarchical but involves complex interactions among multiple oscillatory components. Thus, the age-related alterations in FAA observed in the present study may reflect changes in the coordination among these oscillators rather than a simple impairment of a single circadian pacemaker [6]. The transient coexistence and eventual fusion of daytime and nocturnal components following RF further illustrate how two circadian systems — the SCN-based light-entrainable oscillator and the feeding-entrainable oscillator — can interact, compete, and re-synchronize to generate coherent behavioral rhythms.

### Age-related attenuation and mechanistic interpretation

In the present study, we used middle-aged mice rather than aged animals. This approach is consistent with previous studies examining age-related changes in circadian function using behavioral paradigms [12], [22]. Therefore, the findings should be interpreted as reflecting age-related changes rather than those associated with advanced aging. Aging is known to reduce the amplitude and precision of circadian rhythms in both the SCN and its downstream oscillators. In rodents, age-related dampening of neuronal synchrony and weakened output signals from the SCN lead to instability of behavioral rhythms and slower re-entrainment to shifted light–dark cycles [21], [22]. The present study extends these findings to food-entrainable rhythms, showing that aging also attenuates the flexibility of the food-entrainable oscillator. The delayed appearance and incomplete merging of FAA in middle-aged mice indicate a decline in the neural plasticity or coupling strength required for the FEO to establish a stable phase relationship with the SCN-driven system.

Similar age-dependent deterioration of feeding-associated rhythms was reported in rats, where corticosterone peaks before feeding were delayed or lost in older animals [23]. Together with the present behavioral data, these results suggest that the capacity of the circadian network to reorganize under new timing cues—whether light or food—is progressively compromised with age. We propose that aging weakens inter-oscillator communication within the circadian hierarchy, leading to prolonged transient phases and reduced entrainability. This decline in temporal plasticity may underlie age-related impairments in metabolic adaptation and behavioral flexibility.

It is also possible that middle-aged mice differ from young-adult mice in circadian clock function prior to the onset of restricted feeding. Previous studies have shown that aging is associated with alterations in circadian organization, including changes in clock gene expression and reduced robustness of circadian rhythms [12]. Such baseline differences in circadian regulation may contribute to the delayed emergence and instability of FAA observed in middle-aged mice in the present study. Therefore, the age-related changes in FAA reported here may reflect not only differences in food-entrained mechanisms but also pre-existing alterations in the circadian system.

### Physiological implications and limitations

The present findings provide behavioral evidence that aging reduces the plasticity of circadian organization and weakens the integration between multiple oscillatory components. Such a decline in coupling may contribute to age-related metabolic and behavioral disorders, including impaired adaptation to feeding schedules or altered energy balance. Recent studies using cellular recording approaches have demonstrated that the food-anticipatory activity rhythm arises from the coordinated action of multiple oscillators outside the SCN [24]. These multi-oscillatory mechanisms may underlie the persistence and gradual realignment of FAA observed in the present study.

Food-anticipatory activity is known to be associated with endocrine and metabolic signals, including preprandial increases in corticosterone and other hormones involved in energy homeostasis. Such physiological signals are thought to contribute to the anticipation of scheduled feeding and may interact with circadian regulatory mechanisms. Although the present study did not directly assess these physiological markers, age-related alterations in endocrine signaling may also contribute to the delayed and unstable FAA observed in middle-aged mice. Future studies incorporating hormonal and metabolic measurements will be important to further clarify the physiological basis of age-related changes in food-anticipatory rhythms.

Finally, several limitations should be acknowledged. First, FAA in this study was assessed through wheel-running activity, which reflects motivational and reward-related factors as well as circadian control. In addition, this method does not capture feeding-related behaviors during the feeding period itself, and therefore may not fully reflect all aspects of food-anticipatory responses.　Second, although our data clearly demonstrate the presence and aging-related alteration of the food-entrainable oscillator, the anatomical substrate of this oscillator remains undetermined. Identifying its location—whether in the hypothalamus or peripheral metabolic tissues—will require future work employing molecular and electrophysiological mapping techniques.

## Conclusions

These findings suggest that age-related changes weaken the synchronizing capacity of the circadian system during transitions from light- to food-entrainment, resulting in delayed and unstable expression of food-anticipatory activity. Understanding how such changes in circadian plasticity affect metabolic homeostasis may provide new insight into the temporal regulation of physiology during the aging process.

## CRediT authorship contribution statement

**Sachi N. Ohno:** Writing – review & editing, Writing – original draft, Methodology, Investigation, Funding acquisition, Formal analysis, Data curation, Conceptualization. **Mitsutaka Sugimura:** Writing – review & editing, Funding acquisition, Data curation. **Wataru Nakamura:** Writing – review & editing, Writing – original draft, Visualization, Validation, Supervision, Project administration, Methodology, Investigation, Funding acquisition, Formal analysis, Data curation, Conceptualization. **Takahiro J. Nakamura:** Writing – review & editing, Validation, Funding acquisition, Data curation. **Hitoshi Uchida:** Writing – review & editing, Data curation. **Nana N. Takasu:** Writing – review & editing, Writing – original draft, Visualization, Validation, Methodology, Investigation, Funding acquisition, Formal analysis, Data curation.

## Funding

This work was supported by the Japan Society for the Promotion of Science (10.13039/501100001691JSPS) KAKENHI (23K09316 to S.O.; 24K09635 to N.N.T.; 19K10046 to W.N.; 24K10029 to T.J.N.; 24K13072 to M.S.) and AMED-CREST (24022159 to T.J.N.).

## Declaration of Competing Interest

The authors declare the following financial interests/personal relationships which may be considered as potential competing interests: Wataru Nakamura reports financial support was provided by Japan Society for the Promotion of Science. Sachi N. Ohno reports financial support was provided by Japan Society for the Promotion of Science. Nana N. Takasu reports financial support was provided by Japan Society for the Promotion of Science. Takahiro J. Nakamura reports financial support was provided by Japan Agency for Medical Research and Development. Mitsutaka Sugimura reports financial support was provided by Japan Society for the Promotion of Science. If there are other authors, they declare that they have no known competing financial interests or personal relationships that could have appeared to influence the work reported in this paper.

## Data Availability

The datasets generated and/or analyzed during the current study are available from the corresponding author upon reasonable request.
