# Supplementary material for: Middle-aged mice show delayed and destabilized food-anticipatory circadian activity under restricted feeding
Source: J Physiol Sci. 2026 Apr 2;76(2):100071. doi: 10.1016/j.jphyss.2026.100071 (PMC13091282; doi:10.1016/j.jphyss.2026.100071)
Supplement: Supplementary file 1 — Supplementary material [file mmc1.docx]

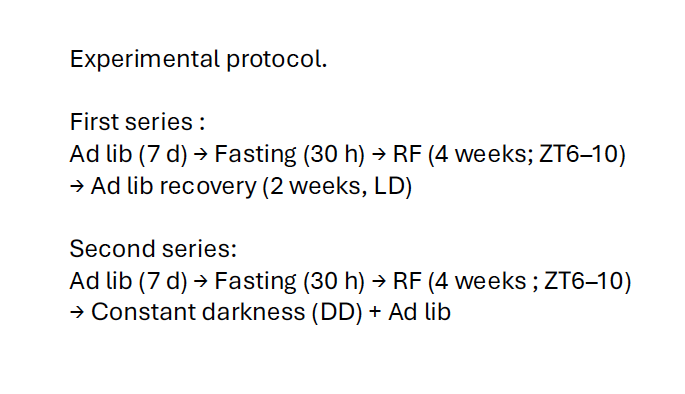


**Supplementary Figure 1. Schematic timeline of the experimental protocol.** Mice were first maintained under ad libitum feeding for 7 days, followed by 30-h fasting and a 4-h restricted feeding (RF) schedule (ZT6–ZT10) for 4 weeks. After RF, animals were returned to ad libitum feeding for 2 weeks under a light–dark (LD) cycle. In a second experimental series, mice underwent an additional RF protocol and were subsequently released into constant darkness (DD) with ad libitum feeding. Wheel-running activity was recorded continuously throughout the experiments.
